# Using a discrete choice experiment to inform the design of programs to promote colon cancer screening for vulnerable populations in North Carolina

**DOI:** 10.1186/s12913-014-0611-4

**Published:** 2014-11-30

**Authors:** Michael P Pignone, Trisha M Crutchfield, Paul M Brown, Sarah T Hawley, Jane L Laping, Carmen L Lewis, Kristen Hassmiller Lich, Lisa C Richardson, Florence KL Tangka, Stephanie B Wheeler

**Affiliations:** Division of General Internal Medicine, University of North Carolina at Chapel Hill, School of Medicine, 5045 Old Clinic Building, CB#7110, Chapel Hill, NC 27599 USA; University of North Carolina at Chapel Hill, Center for Health Promotion and Disease Prevention, University of North Carolina at Chapel Hill, 1700 Martin Luther King Jr. Boulevard, CB#7426, Chapel Hill, NC 27599-7426 USA; University of California - Merced, Health Sciences Research Institute, 5200 North Lake Road, Merced, CA 95343 USA; Division of General Medicine, University of Michigan, 1500 E. Medical Center Drive, Ann Arbor, MI 48109 USA; University of North Carolina at Chapel Hill, Translational and Clinical Sciences Institute, 160 N. Medical Drive Brinkhous-Bullit, 2nd Floor CB#7064, Chapel Hill, USA; Division of General Internal Medicine, Department of Medicine, University of Colorado School of Medicine, Mail Stop B180 Academic Office 1, Room 8415 12631 E. 17th Ave, Aurora, CO 80045 USA; University of North Carolina at Chapel Hill, Gillings School of Global Public Health, Department of Health Policy and Management, University of North Carolina at Chapel Hill, 135 Dauer Drive, CB#7411, McGavran Greenberg Hall, Chapel Hill, NC 27599-7411 USA; Division of Cancer Prevention and Control, Centers for Disease Control and Prevention Centers for Disease Control and Prevention, 1600 Clifton Road, Atlanta, GA 30333 USA

**Keywords:** Early detection of cancer, Colorectal neoplasms, Vulnerable populations, Rural population, Choice behavior, Decision making, Economics, Behavioral, Questionnaires, Health policy

## Abstract

**Background:**

Screening for colorectal cancer (CRC) is suboptimal, particularly for vulnerable populations. Effective intervention programs are needed to increase screening rates. We used a discrete choice experiment (DCE) to learn about how vulnerable individuals in North Carolina value different aspects of CRC screening programs.

**Methods:**

We enrolled English-speaking adults ages 50–75 at average risk of CRC from rural North Carolina communities with low rates of CRC screening, targeting those with public or no insurance and low incomes. Participants received basic information about CRC screening and potential program features, then completed a 16 task DCE and survey questions that examined preferences for four attributes of screening programs: testing options available; travel time required; money paid for screening or rewards for completing screening; and the portion of the cost of follow-up care paid out of pocket. We used Hierarchical Bayesian methods to calculate individual-level utilities for the 4 attributes’ levels and individual-level attribute importance scores. For each individual, the attribute with the highest importance score was considered the most important attribute. Individual utilities were then aggregated to produce mean utilities for each attribute. We also compared DCE-based results with those from direct questions in a post-DCE survey.

**Results:**

We enrolled 150 adults. Mean age was 57.8 (range 50–74); 55% were women; 76% White and 19% African-American; 87% annual household income under $30,000; and 51% were uninsured. Individuals preferred shorter travel; rewards or small copayments compared with large copayments; programs that included stool testing as an option; and greater coverage of follow-up costs. Follow-up cost coverage was most frequently found to be the most important attribute from the DCE (47%); followed by test reward/copayment (33%). From the survey, proportion of follow-up costs paid was most frequently cited as most important (42% of participants), followed by testing options (32%). There was moderate agreement (45%) in attribute importance between the DCE and the single question in the post-DCE survey.

**Conclusions:**

Screening test copayments and follow-up care coverage costs are important program characteristics in this vulnerable, rural population.

**Electronic supplementary material:**

The online version of this article (doi:10.1186/s12913-014-0611-4) contains supplementary material, which is available to authorized users.

## Background

Screening reduces colorectal cancer (CRC) incidence and mortality, and is cost-effective [1,2]. Unfortunately, CRC screening is underutilized, particularly in vulnerable populations, defined as those with low incomes, inadequate or no health insurance, or those who live far from health care facilities [3-6]. Data from national surveys suggest that over a third of US adults ages 50–75 are not up to date with CRC screening; levels are even lower for vulnerable populations: for example, fewer than 50% of those with no insurance report being up to date with CRC screening [6].

The US government’s Healthy People 2020 goals set a target of having 70.5% of eligible adults up to date with CRC screening by 2020 [7]. Effective programs to reach vulnerable populations (and increase their rates of CRC screening) are essential to bring the benefits of CRC screening to the full US population and reach national objectives. Increasing CRC screening is also an important part of payer-based healthcare quality improvement and pay for performance efforts.

Several types of interventions, including small media, patient and provider reminders, and possibly financial incentives are effective in increasing screening, and some of these interventions have been tested in vulnerable populations [8]. However, these effective interventions have not been widely disseminated [6]. Reasons for failure of dissemination may include intervention complexity, costs of intervention, challenges in scaling, or difficulty in choosing among potentially effective interventions. Policy makers and payers may need to decide among potential options for programs to increase CRC screening among vulnerable populations, including the option of maintaining the status quo.

For a screening program to be effective, it must be acceptable to the targeted population, easily accessible, and readily available at an acceptable price. Information on the public's preferences between types of screening programs will aid policy makers in designing, implementing and disseminating effective programs. One method for informing decisions about potential options for increasing CRC screening among vulnerable populations is to elicit feedback from members of vulnerable groups about the features of potential programs.

Discrete choice experiment (DCE) is one potentially valuable method for eliciting such feedback. DCE presupposes that a “product” like a screening promotion program can be described in terms of its features or “attributes” and that by understanding the different features and how people value them, effective programs can be designed and implemented to address the needs of the population [9]. Although stated preference methods, including DCE, have been used to understand patient preferences for different CRC screening tests [10-12], to our knowledge, DCE has not been used in the design of programs to increase CRC screening among vulnerable populations in the US, such as those with low income or those who are under- or uninsured. As such, we designed and performed a DCE among members of vulnerable populations in North Carolina to provide information to assist policymakers in the design and implementation of programs to promote CRC screening.

## Methods

### Overview

We identified rural North Carolina counties with low CRC screening rates and elevated CRC mortality by linking North Carolina Cancer registry data to Medicare and Medicaid claims data using demonstrated methods [13,14]. Three counties in western North Carolina were selected for further study. We then recruited and surveyed English-speaking, adults ages 50–75 at average risk for CRC about their preferences and values regarding possible CRC screening programs.

### Ethical considerations

This study was approved by the Institutional Review Board at the University of North Carolina at Chapel Hill.

### Survey content

The study team designed a paper-based survey based on our prior research [10,11], existing literature [15-19], and pre-testing feedback (See Additional file 1). The survey began with general information about colon cancer and features of colon cancer screening programs and asked participants to complete a DCE, a rating task and several additional questions. We used the term “colon cancer” rather than “colorectal cancer” to ensure understanding among the target population.

### Discrete choice experiments

In a DCE, individuals are asked to choose between hypothetical alternatives described by a set of attributes and levels. The levels of the attributes are varied systematically in a series of choice tasks where participants are asked to select an option that they most prefer. Responses to the choices are modeled to provide quantitative information about the relative value participants attach to different levels of the attributes being considered [20].

### Selection of key attributes and levels

We developed four key attributes of CRC screening programs from the literature and prior research, which include, testing options, travel time, money paid for screening, and the portion of the cost of follow-up care paid out of pocket [10,11,16-19].

We developed a plausible range of levels for each attribute. We framed the testing options attribute by employing four levels: two without a choice (stool test only; colonoscopy only) and two options with a choice (choice of stool test or colonoscopy; choice of stool test, colonoscopy, or CT colonography).We did not include sigmoidoscopy as one of the testing options within a program because our formative research suggested it was not readily available in the study area.

Travel time options ranging from “no travel” to “1 hour + .” were based on distances to nearest endoscopy facilities from our formative work [13]. These times are suitable for the rural geographic region where the study occurred and allowed individuals who may have difficulty conceptualizing distance (e.g. individuals who do not drive) to state their preference.

Money paid for screening was framed to include rewards and costs (copayments) within the same attribute, ranging from a $100 reward to a $1000 cost, with the highest amount of cost designed to simulate the cost of colonoscopy screening without insurance coverage. [21].

We represented out-of-pocket follow-up costs using percentages rather than absolute costs because it is impossible to know what the out-of-pocket follow-up costs might be with the selection of any given screening test. The outcome of the initial screening test and subsequent testing or treatment utilized, factor into the total out-of-pocket costs, and will differ for each individual. Given the uncertainty and difficulty in making a choice among these levels, we decided to frame the levels in a way that is likely to be familiar to individuals - as insurance coverage. The levels ranged from no coverage (100% paid by the person) to a 20% copayment, to full coverage (0% out of pocket expense).

### Development of the DCE

**Figure 1:**
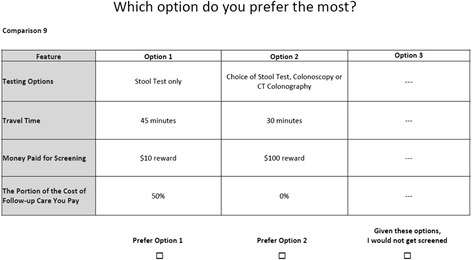
**An example discrete choice experiment task.**

We used Sawtooth Software Version 8 to design a balanced and efficient set of 16 choice tasks (with 1 dominant task): this number of questions has been shown in past work to be feasible for participants to complete [10,11]. Each choice was comprised of two active screening options and a fixed “opt-out” option (“Given these options, I would not get screened”). A sample choice task is included in Figure 1. Our study followed the International Society for Pharmacoeconomics and Outcomes Research (ISPOR) Guidelines [22] for DCE design.

### Post-DCE questions

Following the DCE, participants completed a rating task where they were asked to rate screening program attributes (on a scale from 0, meaning not important at all, to 5, very important, which were converted to a 1 to 6 scale for analysis). Participants were then asked to answer which single program attribute was most important- we called this the “directly selected most important attribute”.

### Pretesting

The survey was pre-tested with 53 individuals in 6 gender-stratified focus groups held in 2 eastern North Carolina counties and 3 western North Carolina counties, from March 28, 2012 to September 7, 2012. Based on participant feedback, the survey was modified to include a table of pictograms comparing CRC screening options, and bulleted, condensed text to improve readability and comprehension. The table of pictograms illustrating screening test options was drawn from evidence-based materials from the National Cancer Institute (http://gutcheck.cancer.gov/screenings/index.html) and from clip art (See Additional file 1).

### Data collection

Participants were recruited from community-based organizations in the region from October 23, 2012 to March 23, 2013. Eligibility criteria included residing in the area of interest (in an identified county or in an adjacent county), being 50 – 75 years of age, having average risk for CRC (i.e. no personal or family history of CRC and no history of inflammatory bowel disease), having a life expectancy of 2+ years, and being able to read English.

Participants were identified and screened for eligibility by one of two methods. Most commonly, individuals learned of the study and called a dedicated phone number, were screened by study personnel, and scheduled an appointment to be surveyed at survey sites, including flea markets, senior centers, discount stores, public housing authorities and medical clinics. Additionally, participants were recruited in-person at survey sites and were screened and administered surveys on a walk-in basis.

To ensure sufficient representation of vulnerable populations within the sample, the study team adopted a purposeful sampling approach for recruitment. We sought to enroll uninsured individuals (at least 50%) or publicly insured individuals (Medicare, Medicaid, Veterans benefits); individuals who were not up-to-date with screening (colonoscopy more than 10 years ago, sigmoidoscopy more than 5 years ago, Fecal Occult Blood Test (FOBT) or Fecal Immunochemical Test (FIT) more than one year ago); individuals who have a low annual household income (< $45,000); and minority groups (African-American and Hispanic participants).

Participants provided consent and were asked to take the survey in semi-private areas. The survey was administered on paper. Each individual was given the option of having the entire survey read aloud by a study team member. Study team members carefully explained how to complete the survey and took the time to explain the DCE process, and went through a practice DCE task to help reduce concerns and uncertainties about how to answer DCE questions. Upon completion of the survey, participants received a $35 gift card.

### Study outcomes

The primary outcomes of interest were the mean utility levels for the four attributes, and the DCE-calculated most important attributes for each individual, based on their individual utility levels. Secondary outcomes of interest include the directly reported single most important attribute and the agreement between the DCE-generated and directly selected most important attribute.

### Sample size determination

Because there is no optimal method for determining the sample size for DCE, we used the method suggested by Johnson to determine that a sample size of 150 participants would yield reasonably precise estimates of utility levels, given the use of 16 choice tasks, two active alternatives, and a maximum number of levels within a single attribute of six [23]. We continued recruitment until we reached this goal.

### Statistical analysis

We performed descriptive analyses with means and proportions using Stata version 12.

The DCE survey responses were analyzed using Choice Based Conjoint (CBC) Hierarchical Bayesian (HB) module in Sawtooth Software to obtain individual-level preferences. The CBC/HB module uses data from the DCE in a mixed effects multivariate, multinomial logit model to estimate the value or “utility” each participant attaches to the different levels of the 4 attributes. The value associated with the “opt-out” choice is expressed as a constant [24].

The Hierarchical Bayesian process used in Sawtooth Software is a two-step process to determine individual-level utilities. First, average utilities are calculated for the full sample of participants, and how much each individual’s responses differ from the average. Secondly, individual utilities are adjusted to an optimal mix of average sample utilities and individual utilities, based on the amount of information provided by participants and the variance in the sample average (Markov Chain Monte Carlo). These individual-level utility estimates are then averaged to give the population mean utilities [24].

Utilities are arbitrarily-scaled, zero-centered values that represent the relative desirability of the levels within each attribute in numerical form: the higher the number the more desirable it is to participants and conversely, the lower (more negative) the number, the less desirable it is to participants [20]. Each participant’s utilities are then used to calculate individual-level attribute importance scores. Attribute importance scores represent the relative importance of the four attributes, given the range of levels employed [20]. Attribute importance scores were calculated for each individual separately and then averaged for mean importance scores.

For each individual, the attribute with the highest importance score from the DCE is considered to be “most important” for that individual. We compared the DCE-based most important attribute with the most important attribute based on a single post-DCE survey question to determine if they represented different information.

## Results

There were 360 individuals who expressed interest in the study and 175 potential participants were found eligible. Twenty-four participants declined participation, 1 did not complete, and 150 individuals completed the survey (85.7%).

Participant characteristics are shown in Table 1. Mean age was 58 years, and 55% were female. Most (76%) were White and 40% had a high school education or less. Participants had low household incomes (87% reported less than $30,000 per year) and 51% were uninsured. Most (76%) had never been screened for CRC.

**Table 1:** **Vulnerable North Carolinians completing CRC screening preference questionnaire (n = 150)**

**Mean age (SD)**	**57.9 (6.2)**
**Gender**	
Female	55.3%
**Race/Ethnicity**	
White	76.0%
African American	19.3%
Hispanic/Latino	2.0%
Other	2.7%
**Education**	
Less than high school graduate	10.7%
High school graduate/GED	29.5%
Some College OR 2-year college Graduate	36.5%
4 Year College graduate or more	23.5%
**Need help with written materials**	
Always	2.0%
Often	2.0%
Sometimes	13.3%
Rarely	24.0%
Never	58.7%
**Income**	
<$30,000	87.4%
$30,000-59,999	12.6%
**Employment**	
Employed	28.2%
Retired	19.5%
Unemployed	21.5%
Disabled	24.2%
Other	6.7%
**CRC screening status**	
Never Screened	76.0%
Ever Screened, Not Up to date	21.3%
Up to Date	2.7%
**Insurance Status** ^**a**^	
Uninsured	50.7%
Private Insurance	7.4%
Medicaid	20.9%
Medicare	31.3%
Medicare Supplement	8.1%
VA/Military Benefits	5.4%

*Abbreviations: CRC* Colorectal Cancer, *SD* Standard Deviation, *GED* General Educational Development, *VA* Veterans Affairs.

^a^Insurance Status categories may overlap, as some individuals are insured by multiple policies or programs.

Mean utility levels are shown in Table 2. The negative constant associated with “Given these options, I would not get screened” suggests a strong overall preference for participation in CRC screening programs. Over 80% of respondents answered the dominant question correctly, suggesting good understanding and engagement in the survey.

**Table 2:** **Discrete choice experiment results: colorectal cancer screening program preferences among vulnerable North Carolinians**

**Attribute**	**Level**	**Mean** ^**a**^ **raw utilities**	**Lower CI**	**Upper CI**	**Mean attribute** ^**b**^ **Importance scores (CI)**
**Testing options**	Stool test only	0.60	0.33	0.87	0.17 (0.16 - 0.18)
	Colonoscopy only	−0.42	−0.72	−0.12	
	Choice: stool test OR Colonoscopy	−0.61	−0.76	−0.47	
	Choice: stool test, Colonoscopy OR CT Colonography	0.43	0.26	0.60	
**Travel time**	No Travel	1.03	0.89	1.17	0.16 (0.15 - 0.17)
	15 minutes	0.92	0.78	1.06	
	30 minutes	0.32	0.23	0.41	
	45 minutes	−0.96	−1.15	−0.77	
	1 hour +	−1.31	−1.46	−1.16	
**Money paid for screening**	$100 Reward	2.50	2.24	2.75	0.33 (0.32 - 0.35)
	$10 Reward	−0.06	−0.23	0.10	
	$0	0.30	0.06	0.53	
	$25 Cost	1.08	0.87	1.29	
	$100 Cost	−0.15	−0.41	0.11	
	$1000 Cost	−3.66	−3.96	−3.36	
**% follow-up care cost you pay**	0%	2.79	2.33	3.25	0.34 (0.32 - 0.36)
	5%	1.68	1.40	1.96	
	20%	0.42	0.30	0.55	
	50%	−1.48	−1.73	−1.23	
	100%	−3.42	−3.84	−2.99	
**None**	Given these options, I would not get screened	−3.19	−3.82	−2.56	n/a

*Abbreviations: CI* Confidence Interval, *CT* Computerized Tomography.

^a^Mean raw utilities indicate the relative desirability of each level within an attribute; the higher the number, the more desirable; the lower the number (the more negative), the less desirable.

^b^The relative importance of each attribute, when the stated levels included are employed. The importance scores sum to 1.0 and can be interpreted as proportions.

As predicted, participants preferred no or shorter travel times compared with longer travel times. They also exhibited strong preferences for the larger ($100) reward for screening and against the highest screening test copayment of $1000. Interestingly, they had few differences in utility among small ($10) rewards, small-moderate copayments ($25- $100), or the lack of a reward or copayment. These findings suggest that individuals would be more likely to be screened if given a large reward (small rewards and copayments are less likely to have a significant impact) and would be discouraged from participating in a program where they had to bear large costs for screening.

The proportion of follow-up costs covered by the screening program was also important: participants strongly preferred full or nearly full coverage of follow-up costs compared with programs in which they would be responsible for 50 or 100% of these costs.

Utility levels for test choices available within the hypothetical screening programs had a less consistent pattern. In general, participants appeared to value having the option of stool test screening, whether as the only testing option or as part of a suite of options that included CT colonography and colonoscopy. In contrast, programs offering colonoscopy as the only option or offering a choice of stool test or colonoscopy were relatively less popular.

Mean attribute importance scores, which reflect the relative importance of the four attributes compared to one another, given the levels of the attributes employed, suggested that the cost variables were relatively more important than testing options or travel time: follow-up care cost had a 34% importance score, followed by money paid for screening (reward or copayment) at 33%. The other attributes, testing options (17%) and travel time (16%), were somewhat less important given the ranges of levels tested.

### Post-DCE survey

In the post-DCE survey, participants’ ratings of the importance of the attributes had some similarities to the DCE results: the cost of follow-up care was rated to be very important (mean 5.5, SD 1.1 on a 1–6 scale), while travel time was rated to be of lower importance (mean 3.4, SD 1.8). In contrast, testing options were considered to be quite important (mean 5.3, SD 1.2), and out of pocket costs or rewards somewhat less so (mean 4.6, SD 1.8).

The most important attribute, based on a single question, was most frequently found to be the costs of follow-up care (42%), followed by testing options (32%), and out of pocket screening test costs or rewards (23%). Only 1.3% of participants considered travel time to be most important.

### Agreement between DCE and survey results

Table 3 shows the agreement between participants’ most important attribute as calculated from the DCE in comparison with their responses to the single question from the post-DCE survey. Overall, 66 of 148 (45%) participants had agreement between these two methods for assessing attribute importance, with most differences coming from differences between methods in the value attributed to testing options compared with costs.

**Table 3:** **Agreement between most importance attribute and most important single question attribute**

**Counts of question responses** ^**a**^					
**DCE most important attribute**					
	**Testing options**	**Travel time**	**Screening rewards/Costs**	**Follow-up costs**	**Totals**
**Single question most important attribute**					
Testing Options	**9**	0	24	15	48
Travel Time	0	**0**	1	1	2
Screening Rewards/Costs	1	1	**17**	16	35
Follow-up Costs	2	0	21	**40**	63
Totals	12	1	63	72	148

*Abbreviation: DCE* Discrete Choice Experiment.

^a^Agreement 66/148 (45%), determined by taking a count of the questions that were answered the same (as indicated by the bold numbers in the diagonal- 66 total) and dividing by the total number of responses (148).

## Discussion

In our survey of vulnerable adults in North Carolina, we found strong overall interest in programs to promote CRC screening. Program costs, both those associated with screening and those related to follow-up costs after initial screening, strongly influenced participants’ preferences. Participants’ responses also seemed to favor programs that included the option of stool testing, which may also reflect concerns about the costs of screening colonoscopy. Somewhat surprisingly, travel time appeared relatively much less important, perhaps reflecting the rural setting for our survey; residents of rural areas may be more accustomed to traveling relatively long distances and times to obtain health care, and hence attribute less relative importance to it.

Our study is the first, to our knowledge, to use DCE to examine features of potential CRC screening programs in the United States (US). Our findings have several potential implications for US health policy and for future research. First, they suggest that in order to maximize uptake and effectiveness, policymakers could encourage the development of programs that account for both initial screening costs and subsequent costs for additional testing (e.g. diagnostic and surveillance colonoscopy) and cancer treatment. The uncertainty associated with the magnitude of the follow-up costs may be a particularly important barrier to overcome.

Secondly, such programs could include the option of stool testing, which can be performed at home and has lower “up-front” effort and costs required compared with screening colonoscopy. Along these lines, Inadomi and colleagues found that uptake of CRC screening in a vulnerable urban population was higher when patients were offered FOBT alone or the option of FOBT or colonoscopy compared with only offering screening colonoscopy [25].

Our work suggests that DCE is a feasible technique for eliciting preferences of vulnerable individuals about potential health promotion policies. Based on prior behavioral economics research [26,27], we expected to see stronger negative preferences for the modest copayments and more strongly positive preferences for the modest reward levels; the lack of such effects suggests that the relatively small monetary copayments or rewards may have little effect themselves on program uptake, at least in comparison with the extreme levels (e.g. those associated with having to pay for screening colonoscopy completely out of pocket). Also, our DCE results differed somewhat from our direct question results, with the DCE suggesting a great deal of importance attached to costs whereas the direct questions also found considerable importance attached to testing options. These results suggest that the different techniques (DCE and direct questions) may have complementary value [10,11].

Our findings should be considered in light of several limitations. Foremost, we examined participants’ stated preferences; their actual behaviors (e.g. actual participation in screening programs) may differ. Secondly, we were only able to test a limited number of attributes and levels while maintaining a feasible and valid survey design; including other attributes or different levels within attributes, may have produced somewhat different results. That said, we believe that the attributes and levels we chose are relevant and well supported in the literature. The participants’ responses about the choice of screening tests available within the screening program were somewhat complex and hard to interpret: they reflected participants’ feeling about the specific tests as well as about the value of having options. It appears participants attached particular value to having stool testing as one of the options, but did not necessarily value more choice simply for the sake of having more options. Moreover, participants received brief, high-quality, evidence-based information about the testing options, but because we were studying preferences for screening programs, we did not evaluate their understanding of the nuances of each test.

We chose to represent out of pocket follow-up costs with percentages rather than absolute amounts because the amounts of out of pocket follow-up costs vary so much according to whether the person always screens negative (and hence has no additional costs), requires diagnostic colonoscopy with or without polypectomy, or is diagnosed with cancer and requires treatment. Given this uncertainty, we sought to simulate how insurance programs address costs, ranging from no coverage (100% paid by the person) to a 20% copayment, to full coverage (0% out of pocket expense). Not surprisingly, this attribute was considered very important, and reinforces the need for policymakers to consider follow-up care costs when designing screening programs.

Our sample size of 150 was sufficient, based on expert opinion, to examine appropriately differentiate overall preferences for the levels tested. However, we cannot exclude that a larger sample may not be able to further differentiate small degrees of difference in preferences. Finally, we collected information from one (rural) region of North Carolina; results from other parts of the state or from other states, may differ.

## Conclusions

Despite these limitations, our results suggest that programs to encourage CRC screening among vulnerable individuals are likely to be most effective if they account for both the direct costs of screening as well as the follow-up costs of additional testing and treatment and if they include a stool test option. In our future work, we will use these findings to compare the cost-effectiveness of different means to increase CRC screening at the state level through organized programs, to help provide information and guidance about efficiency as well as potential effectiveness.

## Additional files

Additional file 1:
**Survey Instrument.** Colon Cancer Screening Information.

## Abbreviations

CT: Computerized tomography
CRC: Colorectal cancer
DCE: Discrete choice experiment
FIT: Fecal immunochemical test
FOBT: Fecal occult blood test
ISPOR: International Society for Pharmacoeconomics and Outcomes Research
SD: Standard deviation
