# Supplementary material for: Using a discrete choice experiment to inform the design of programs to promote colon cancer screening for vulnerable populations in North Carolina
Source: BMC Health Serv Res. 2014 Nov 30;14:611. doi: 10.1186/s12913-014-0611-4 (PMC4267137; doi:10.1186/s12913-014-0611-4)
Supplement: Additional file 1: — Survey Instrument. Colon Cancer Screening Information. [file 12913_2014_611_MOESM1_ESM.docx]

Additional file 1: Survey Instrument

ID # ______

Start ______

Finish ______

Admin ID____

**Colon Cancer Screening Information**

- The **colon** is the large intestine or bowel.
- Colon cancer is found in both men and women and is more common after age 50.
- It is recommended that adults ages 50-75 have some form of screening for colon cancer.
- **Screening** means checking for the condition before it causes any symptoms.
- There are several ways to be screened for colon cancer, and no one way is clearly best for everyone.
- People have a choice to be screened or not.
- Your answers to the questions in this survey will help us learn more about the information men and women need to make decisions about colon cancer screening.
- Some of the questions may seem very similar to each other. That’s OK – we simply want to know what you think. To learn how people view different parts of the decision, we often need to ask similar questions in different ways.

**Before we go on, here are some important facts about colon cancer:**

- Screening for colon cancer beginning at age 50 reduces the chance of developing colon cancer or dying from it.
- The lifetime chance of getting colon cancer is about 6 out of 100.
- The lifetime chance of dying from colon cancer is about 3 out of 100.
- There are several good ways to be screened for colon cancer. Each of the different ways of screening for colon cancer, if used regularly, reduces the chances of both getting and dying from colon cancer by about 50% (half).
- The best colon cancer screening option for you depends on how you feel about different features of screening programs.

**Colon Cancer Screening Program Features**

We are studying 4 different features of colon cancer screening programs. After reading about the 4 different features below, you will be asked questions. Please answer the questions to the best of your ability.

**The 4 program features are:**

1. **Testing Options**
2. **Travel Time**
3. **Money Paid for Screening**
4. **The Portion of the Cost of Follow-up Care You Pay**
5. **Testing Options**: This feature is about the type of test options that are available for you to select from. The options include stool test, colonoscopy, CT colonography or combinations of the three tests.

**Stool Test**

-
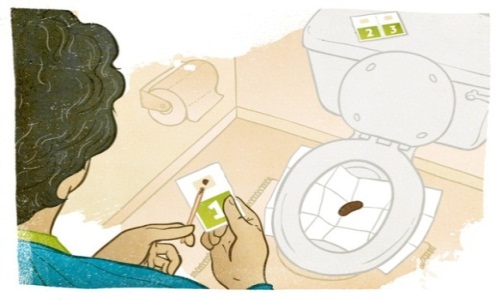
Completed at home with a test kit.
- Requires taking stool samples and mailing the samples to a lab to check for blood.
- If test shows blood in your stool, you will need a colonoscopy.
- Completed in a medical facility.

**Colonoscopy**

-
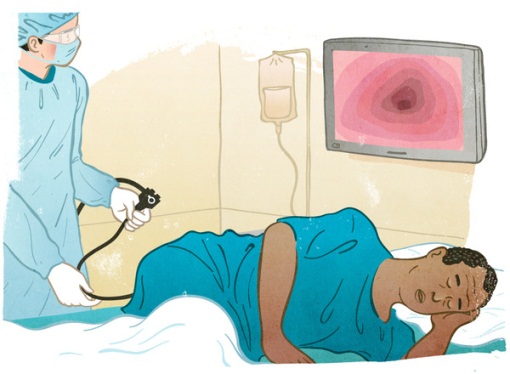
Requires cleansing the stool from your bowels using medicine.
- You will be given medicine to make you sleepy before the test.
- A tube will be inserted into your rectum to check for polyps (abnormal growths) in your bowel.
- If polyps are found they will be removed.
- You may experience some discomfort during the test.
- Companion is required to drive you home.
- 24 hours after the test you can start normal activities again.

- Completed in a medical facility.

**CT Colonography**

-
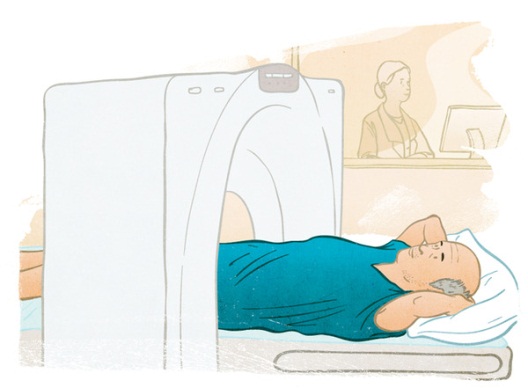
Requires cleansing the stool from your bowels using medicine.
- You will not be given medicine to make you sleepy.
- A small tube will be inserted into your rectum to put air into your bowels.

- You will be asked to lie still on a table for several minutes.
- The test allows x-rays and computers to make a picture of your colon.
- If the test shows a problem you will have to have a colonoscopy.
- You may experience some discomfort.
- A companion is not required to drive you home.
- 1 hour after the test you can start normal activities again.

1. **Travel Time**:

- This feature is about the time it will take you to get to and from a screening test location or time it takes to pick up a home screening kit.
- It includes time you spend driving in a car, waiting on and taking a bus, or waiting on and taking a train.
- Travel time ranges from no travel to 1 hour.
- All things being equal, a shorter travel time is better than a longer travel time.

1. **Money Paid for Screening:**

- This feature is about how much you will have to pay for a screening test (cost) OR how much money is given to you (reward) for having a screening test.
- Costs range from $0 to $1000. Lower costs are better than higher costs.
- Rewards range from $10 to $100. Larger rewards are better than smaller rewards.
- All other things being equal, a reward is better than a cost.

1. **The Portion of the Cost of Follow-up Care You Pay:**

- This feature is about how much you will have to pay for follow-up tests and/or medical treatment if a screening test finds a problem.
- The costs you will have to pay range from 0% to 100% of the costs of follow up care.
- All things being equal, paying 0% is better than paying 100% of the costs of follow up care.
- As an example, if follow-up care costs are $1000, then the portion you pay would be as follows:

0% of $1,000 = $0

5% of $1,000= $50

20% of $1,000= $200

50% of $1,000= $500

100% of $1,000= $1,000

*Images on preceding page are from the National Institutes of Health Gutcheck Project

<http://gutcheck.nci.nih.gov/>

**Pre-Test Questions**

**True or False**

Please **Circle** True or False for the following questions:

1. Getting screened for colon cancer reduces the risk for getting and dying from colon cancer by half.

True False

1. When a person has a colonoscopy they need someone to drive them home.

True False

1. If a stool test or CT colonography test shows a problem, a person will have to have a colonoscopy.

True False

**Part 1: Colon Cancer Screening Comparisons**

**Instructions**

You will see a series of comparisons in this part of the survey. Each comparison shows a choice between options.

- - Each option is made up of different combinations of the 4 colon cancer screening features you just read about.
  - You should assume that the options do not differ in any other ways besides those described in the feature descriptions.
  - Please think about each of the features individually.
  - **These combinations of features of colon cancer screening programs are not necessarily real options.** We are asking you about these possible options to help us learn about what features or groups of features are most important to you.

With each comparison, you will be asked to consider the options and pick which one seems like it would be the best option for you. Remember, **there are no right or wrong answers.** We just want to know which option you prefer.

If you do not like either of the two options, you may decide to select the option, “Given these options, I would not get screened”.

**Please Wait for Further Instruction**

**Practice Comparison: Imagine you are shopping for a used car.**

| **Feature** | **Car 1** | **Car 2** | **Neither Car** |
| --- | --- | --- | --- |
| **Color** | Red | Blue | --- |
| **Make** | Ford | Nissan | --- |
| **Miles** | 155,539 | 39,987 | --- |
| **Number of Doors** | 4 | 2 | --- |
| **Model Year** | 2007 | 1998 | --- |
| **Cost** | $7,200 | $3,982 | --- |
|  | **Prefer Car 1** | **Prefer Car 2** | **Given these options,**  **I would not buy a car.** |
|  | 🞎 | 🞎 | 🞎 |

| **Comparison 1** |  | |  | |  |  |
| --- | --- | --- | --- | --- | --- | --- |
| **Feature** | | **Option 1** | | **Option 2** | | **Option 3** |
| **Testing Options** | | Choice of Stool Test, Colonoscopy or  CT Colonography | | Stool Test only | | --- |
| **Travel Time** | | 1 hour or more | | 30 minutes | | --- |
| **Money Paid for Screening** | | $100 reward | | $25 cost | | --- |
| **The Portion of the Cost of Follow-up Care You Pay** | | 5% | | 100% | | --- |

|  | **Prefer Option 1** | **Prefer Option 2** | **Given these options,**  **I would not get screened** |
| --- | --- | --- | --- |
|  | 🞎 | 🞎 | 🞎 |

| **Comparison 2** |  | |  | |  |  |
| --- | --- | --- | --- | --- | --- | --- |
| **Feature** | | **Option 1** | | **Option 2** | | **Option 3** |
| **Testing Options** | | Colonoscopy only | | Colonoscopy only | | --- |
| **Travel Time** | | 15 minutes | | 15 minutes | | --- |
| **Money Paid for Screening** | | $100 cost | | $1000 cost | | --- |
| **The Portion of the Cost of Follow-up Care You Pay** | | 20% | | 5% | | --- |

|  | **Prefer Option 1** | **Prefer Option 2** | **Given these options,**  **I would not get screened** |
| --- | --- | --- | --- |
|  | 🞎 | 🞎 | 🞎 |

| **Comparison 3** |  | |  | |  |  |
| --- | --- | --- | --- | --- | --- | --- |
| **Feature** | | **Option 1** | | **Option 2** | | **Option 3** |
| **Testing Options** | | Choice of Stool Test, Colonoscopy or  CT Colonography | | Colonoscopy only | | --- |
| **Travel Time** | | No travel required | | 1 hour or more | | --- |
| **Money Paid for Screening** | | $100 cost | | $10 reward | | --- |
| **The Portion of the Cost of Follow-up Care You Pay** | | 50% | | 100% | | --- |

|  | **Prefer Option 1** | **Prefer Option 2** | **Given these options,**  **I would not get screened** |
| --- | --- | --- | --- |
|  | 🞎 | 🞎 | 🞎 |

| **Comparison 4** |  | |  | |  |  |
| --- | --- | --- | --- | --- | --- | --- |
| **Feature** | | **Option 1** | | **Option 2** | | **Option 3** |
| **Testing Options** | | Stool Test only | | Choice of Stool Test or Colonoscopy | | --- |
| **Travel Time** | | No travel required | | 30 minutes | | --- |
| **Money Paid for Screening** | | $10 reward | | $1000 cost | | --- |
| **The Portion of the Cost of Follow-up Care You Pay** | | 0% | | 5% | | --- |

|  | **Prefer Option 1** | **Prefer Option 2** | **Given these options,**  **I would not get screened** |
| --- | --- | --- | --- |
|  | 🞎 | 🞎 | 🞎 |

| **Comparison 5** |  | |  | |  |  |
| --- | --- | --- | --- | --- | --- | --- |
| **Feature** | | **Option 1** | | **Option 2** | | **Option 3** |
| **Testing Options** | | Choice of Stool Test, Colonoscopy or  CT Colonography | | Choice of Stool Test or Colonoscopy | | --- |
| **Travel Time** | | 45 minutes | | 15 minutes | | --- |
| **Money Paid for Screening** | | $25 cost | | $100 reward | | --- |
| **The Portion of the Cost of Follow-up Care You Pay** | | 20% | | 50% | | --- |

|  | **Prefer Option 1** | **Prefer Option 2** | **Given these options,**  **I would not get screened** |
| --- | --- | --- | --- |
|  | 🞎 | 🞎 | 🞎 |

| **Comparison 6** |  | |  | |  |  |
| --- | --- | --- | --- | --- | --- | --- |
| **Feature** | | **Option 1** | | **Option 2** | | **Option 3** |
| **Testing Options** | | Stool Test only | | Colonoscopy only | | --- |
| **Travel Time** | | 1 hour or more | | 45 minutes | | --- |
| **Money Paid for Screening** | | $0 | | $100 cost | | --- |
| **The Portion of the Cost of Follow-up Care You Pay** | | 20% | | 5% | | --- |

|  | **Prefer Option 1** | **Prefer Option 2** | **Given these options,**  **I would not get screened** |
| --- | --- | --- | --- |
|  | 🞎 | 🞎 | 🞎 |

| **Comparison 7** |  | |  | |  |  |
| --- | --- | --- | --- | --- | --- | --- |
| **Feature** | | **Option 1** | | **Option 2** | | **Option 3** |
| **Testing Options** | | Colonoscopy only | | Choice of Stool Test, Colonoscopy or  CT Colonography | | --- |
| **Travel Time** | | 30 minutes | | No travel required | | --- |
| **Money Paid for Screening** | | $0 | | $1000 cost | | --- |
| **The Portion of the Cost of Follow-up Care You Pay** | | 50% | | 100% | | --- |

|  | **Prefer Option 1** | **Prefer Option 2** | **Given these options,**  **I would not get screened** |
| --- | --- | --- | --- |
|  | 🞎 | 🞎 | 🞎 |

| **Comparison 8** |  | |  | |  |  |
| --- | --- | --- | --- | --- | --- | --- |
| **Feature** | | **Option 1** | | **Option 2** | | **Option 3** |
| **Testing Options** | | Choice of Stool Test or Colonoscopy | | Stool Test only | | --- |
| **Travel Time** | | No travel required | | 15 minutes | | --- |
| **Money Paid for Screening** | | $100 cost | | $25 cost | | --- |
| **The Portion of the Cost of Follow-up Care You Pay** | | 20% | | 0% | | --- |

|  | **Prefer Option 1** | **Prefer Option 2** | **Given these options,**  **I would not get screened** |
| --- | --- | --- | --- |
|  | 🞎 | 🞎 | 🞎 |

| **Comparison 9** |  | |  | |  |  |
| --- | --- | --- | --- | --- | --- | --- |
| **Feature** | | **Option 1** | | **Option 2** | | **Option 3** |
| **Testing Options** | | Stool Test only | | Choice of Stool Test, Colonoscopy or  CT Colonography | | --- |
| **Travel Time** | | 45 minutes | | 30 minutes | | --- |
| **Money Paid for Screening** | | $10 reward | | $100 reward | | --- |
| **The Portion of the Cost of Follow-up Care You Pay** | | 50% | | 0% | | --- |

|  | **Prefer Option 1** | **Prefer Option 2** | **Given these options,**  **I would not get screened** |
| --- | --- | --- | --- |
|  | 🞎 | 🞎 | 🞎 |

| **Comparison 10** |  | |  | |  |  |
| --- | --- | --- | --- | --- | --- | --- |
| **Feature** | | **Option 1** | | **Option 2** | | **Option 3** |
| **Testing Options** | | Choice of Stool Test or Colonoscopy | | Colonoscopy only | | --- |
| **Travel Time** | | 15 minutes | | 1 hour or more | | --- |
| **Money Paid for Screening** | | $100 cost | | $25 cost | | --- |
| **The Portion of the Cost of Follow-up Care You Pay** | | 100% | | 5% | | --- |

|  | **Prefer Option 1** | **Prefer Option 2** | **Given these options,**  **I would not get screened** |
| --- | --- | --- | --- |
|  | 🞎 | 🞎 | 🞎 |

| **Comparison 11** |  | |  | |  |  |
| --- | --- | --- | --- | --- | --- | --- |
| **Feature** | | **Option 1** | | **Option 2** | | **Option 3** |
| **Testing Options** | | Stool Test only | | Choice of Stool Test or Colonoscopy | | --- |
| **Travel Time** | | 15 minutes | | 1 hour or more | | --- |
| **Money Paid for Screening** | | $0 | | $1000 cost | | --- |
| **The Portion of the Cost of Follow-up Care You Pay** | | 0% | | 100% | | --- |

|  | **Prefer Option 1** | **Prefer Option 2** | **Given these options,**  **I would not get screened** |
| --- | --- | --- | --- |
|  | 🞎 | 🞎 | 🞎 |

| **Comparison 12** |  | |  | |  |  |
| --- | --- | --- | --- | --- | --- | --- |
| **Feature** | | **Option 1** | | **Option 2** | | **Option 3** |
| **Testing Options** | | Choice of Stool Test, Colonoscopy or  CT Colonography | | Choice of Stool Test or Colonoscopy | | --- |
| **Travel Time** | | 15 minutes | | 1 hour or more | | --- |
| **Money Paid for Screening** | | $0 | | $1000 cost | | --- |
| **The Portion of the Cost of Follow-up Care You Pay** | | 5% | | 50% | | --- |

|  | **Prefer Option 1** | **Prefer Option 2** | **Given these options,**  **I would not get screened** |
| --- | --- | --- | --- |
|  | 🞎 | 🞎 | 🞎 |

| **Comparison 13** |  | |  | |  |  |
| --- | --- | --- | --- | --- | --- | --- |
| **Feature** | | **Option 1** | | **Option 2** | | **Option 3** |
| **Testing Options** | | Stool Test only | | Colonoscopy only | | --- |
| **Travel Time** | | 30 minutes | | 45 minutes | | --- |
| **Money Paid for Screening** | | $10 reward | | $100 reward | | --- |
| **The Portion of the Cost of Follow-up Care You Pay** | | 20% | | 100% | | --- |

|  | **Prefer Option 1** | **Prefer Option 2** | **Given these options,**  **I would not get screened** |
| --- | --- | --- | --- |
|  | 🞎 | 🞎 | 🞎 |

| **Comparison 14** |  | |  | |  |  |
| --- | --- | --- | --- | --- | --- | --- |
| **Feature** | | **Option 1** | | **Option 2** | | **Option 3** |
| **Testing Options** | | Choice of Stool Test, Colonoscopy or  CT Colonography | | Choice of Stool Test or Colonoscopy | | --- |
| **Travel Time** | | 15 minutes | | No travel required | | --- |
| **Money Paid for Screening** | | $10 reward | | $25 cost | | --- |
| **The Portion of the Cost of Follow-up Care You Pay** | | 50% | | 0% | | --- |

|  | **Prefer Option 1** | **Prefer Option 2** | **Given these options,**  **I would not get screened** |
| --- | --- | --- | --- |
|  | 🞎 | 🞎 | 🞎 |

| **Comparison 15** |  | |  | |  |  |
| --- | --- | --- | --- | --- | --- | --- |
| **Feature** | | **Option 1** | | **Option 2** | | **Option 3** |
| **Testing Options** | | Colonoscopy only | | Stool Test only | | --- |
| **Travel Time** | | 1 hour or more | | No travel required | | --- |
| **Money Paid for Screening** | | $100 cost | | $0 | | --- |
| **The Portion of the Cost of Follow-up Care You Pay** | | 0% | | 5% | | --- |

|  | **Prefer Option 1** | **Prefer Option 2** | **Given these options,**  **I would not get screened** |
| --- | --- | --- | --- |
|  | 🞎 | 🞎 | 🞎 |

| **Comparison 16** |  | |  | |  |  |
| --- | --- | --- | --- | --- | --- | --- |
| **Feature** | | **Option 1** | | **Option 2** | | **Option 3** |
| **Testing Options** | | Choice of Stool Test or Colonoscopy | | Choice of Stool Test, Colonoscopy or  CT Colonography | | --- |
| **Travel Time** | | 45 minutes | | 30 minutes | | --- |
| **Money Paid for Screening** | | $100 reward | | $1000 cost | | --- |
| **The Portion of the Cost of Follow-up Care You Pay** | | 20% | | 100% | | --- |

|  | **Prefer Option 1** | **Prefer Option 2** | **Given these options,**  **I would not get screened** |
| --- | --- | --- | --- |
|  | 🞎 | 🞎 | 🞎 |

|  |  |  |  |
| --- | --- | --- | --- |

**Part 2: Direct Choices**

Please answer the questions to the best of your ability. Remember, **there are no right or wrong answers**. We just want to know what you think.

| 1. | Which **ONE** feature of colon cancer screening is most important to you? Please check only one. | | |
| --- | --- | --- | --- |
|  |  | |  |
|  |  | |  |
|  |  | | **Testing Options**  What type of test options are available to choose from. |
|  |  | | **Travel Time**  The time it will take to get to and from a screening test |
|  | |  | **Money Paid for Screening**  How much you will have to pay or how much you will be paid for having a screening test |
|  | |  | **The Portion of the Cost of Follow-up Care You Pay**  How much you will have to pay for follow-up testing and/or medical treatment if a screening test detects a problem. |

| 2. Which **ONE** of the following options is most important to you in a colon cancer  screening program? | | | | | | |
| --- | --- | --- | --- | --- | --- | --- |
|  |  | | |  |  |  |
|  |  |  |  | Being able to choose between different colon cancer screening tests |  |  |
|  |  |  |  | Being able to have the stool test as a screening test |  |  |
|  |  |  |  | Being able to have colonoscopy as a screening test |  |  |
|  |  |  |  | None of the above: I do not intend to be screened for colon cancer |  |  |
|  |  | |  |  |  |  |

| **Part 3: Rating Task**  Please rate each item on a scale of 0 to 5 about how important these items are to you in a colon cancer screening program. **0 is** **NOT AT ALL IMPORTANT** and **5 is VERY IMPORTANT to you.** You may only check one box per question. | | | | | | |
| --- | --- | --- | --- | --- | --- | --- |
|  | NOT AT ALL IMPORTANT |  |  |  |  | VERY IMPORTANT |
|  | **0** | **1** | **2** | **3** | **4** | **5** |
| 1. Testing Options |  |  |  |  |  |  |
| 1. Travel Time |  |  |  |  |  |  |
| 1. Money Paid for Screening |  |  |  |  |  |  |
| 1. The Portion of the Cost of Follow-up Care You Pay |  |  |  |  |  |  |
|  |  |  |  |  |  |  |

**Part 4: Ranking Task**

Out of the list of features below (A-D) **what are the 3 most important features to you in order of importance?**

**Please enter the letter for your first, second, and third most important feature choices in the boxes below.**

|  |  | | |  |
| --- | --- | --- | --- | --- |
| 1. **First most important feature:** |  |  |  |  |
|  |  |  |  |  |
| 1. **Second most important feature:** |  |  |  |  |
|  |  |  |  |  |
| 1. **Third most important feature:** |  |  |  |  |
| 1. Testing Options | | | | |
|  | | | | |
| 1. Travel Time | | | | |
|  | | | | |
| 1. Money Paid for Screening | | | | |
|  | | | | |
| 1. The Portion of the Cost of Follow-up Care You Pay | | | | |
|  | | | | |

**Part 5: Information about You**

| 1. Are you   Male  Female   1. In which North Carolina County do you reside? Pick **only ONE** option that best describes where you live.  \|  \| Rutherford \| \| --- \| --- \| \|  \| McDowell \| \|  \| Madison \| \|  \| Other, Please Specify__________ \|  1. What is your age? ____________ 2. Pick **ONE** option that best describes you:  \|  \| White/Caucasian \| \| --- \| --- \| \|  \| Black/African-American \| \|  \| Asian/Pacific Islander \| \|  \| Hispanic/Latino \| \|  \| Other, Please Specify____________ \|  1. What is the highest grade you completed in school? Pick only **ONE** option.  \|  \| 7^th^ grade or lower \| \| --- \| --- \| \|  \| 8^th^ through 11^th^ \| \|  \| High school graduate or GED \| \|  \| Some college or vocational school \| \|  \| 2-year college degree \| \|  \| 4-year college degree \| \|  \| Professional or graduate degree \| |
| --- | --- | --- | --- | --- | --- | --- | --- | --- | --- | --- | --- | --- | --- | --- | --- | --- | --- | --- | --- | --- | --- | --- | --- | --- | --- | --- | --- | --- | --- | --- | --- | --- |

1. What is your annual household income? Pick only **ONE** option.

|  |  | | | $0 to $14,999 | | |
| --- | --- | --- | --- | --- | --- | --- |
|  |  | | | $15,000 to $29,999 | | |
|  |  | | | $30,000 to $44,999 | | |
|  |  | | | $45,000 to $59,999 | | |
|  |  | | | $60,000 to $74,999 | | |
|  |  | | | $75,000 to $89,999 | | |
|  |  | | | $90,000 or greater | | |
| 1. What is your current employment status?   Pick only **ONE** option. |  |  |  |  |  |  |
|  |  |  | Employed Full Time |  |  |  |
|  |  |  | Employed Part-Time  Self-Employed  Unemployed and actively seeking work |  |  |  |
|  |  |  | Unemployed and not actively seeking work |  |  |  |
|  |  |  | Retired |  |  |  |
|  |  |  | Disabled |  |  |  |
|  |  |  | Other ____________________________ |  |  |  |

1. Do you live on a fixed income (Social Security or Pension)?

Yes

No

1. Do you have private health insurance (such as Blue Cross Blue Shield, United Healthcare or Humana)?

Yes

No

1. Do you have Medicaid?

Yes

No

1. Do you have Medicare?

Yes

No

1. Do you have a Medicare Supplement Policy?

Yes

No

1. Do you have VA or Military medical benefits?

Yes

No

1. Has anyone in your immediate family ever had colon cancer (Immediate family means your mother, father, brother, or sister).

Yes

No

1. Do you know anyone other than your immediate family who has or has had colon cancer?

Yes

No

1. Have you completed a STOOL TEST, also called an FOBT test? *This test requires a series of stool samples to be taken and sent in to a laboratory for testing.*

*.*

Yes, I have completed a STOOL TEST within the last

twelve months.

Yes, I completed a STOOL TEST more than TWELVE

MONTHS ago.

No, I have NOT completed a STOOL TEST.

I Don’t know

1. Have you had a SIGMOIDOSCOPY? *This test must be conducted either in a hospital or doctor’s office. A lighted tube is inserted into the rectum and is used to view the lower part of the colon. You are not given anything to make you sleepy for this test.*

Yes, I have had a SIGMOIDOSCOPY within the last FIVE YEARS.

Yes, I had a SIGMOIDOSCOPY over FIVE YEARS ago.

No, I have NOT had a SIGMOIDOSCOPY.

I Don’t know

1. Have you had a COLONOSCOPY? *This test must be conducted in a medical facility. A lighted tube is inserted into the rectum and used to view the entire colon. You are usually given something* *to make you sleepy for this test.*

Yes, I have had a COLONOSCOPY within the last TEN YEARS.

Yes, I had a COLONOSCOPY over TEN YEARS ago.

No, I have NOT had a COLONOSCOPY.

I Don’t know

1. Have you had a CT COLONOGRAPHY? *This test is conducted in a medical facility. A small tube is inserted into your rectum to put air into your bowels. A CT Scanner uses Xrays to take pictures of your colon. You are usually not given something to make you sleepy for this test.*

Yes, I have had a CT COLONOGRAPHY within the last TEN YEARS.

Yes, I had a CT COLONOGRAPHY over TEN YEARS ago.

No, I have NOT had a CT COLONOGRAPHY

I Don’t know

1. Has a SIGMOIDOSCOPY or COLONOSCOPY shown polyps in your colon? *A polyp is a pre-cancerous growth inside your bowel.*

Yes

No

I Don’t know

1. Compared to other people your age, how do you rate your own risk of getting colon cancer?

Much Lower than average

Lower than average

About average

Higher than average

Much higher than average

1. How often do you need someone to help you when you read instructions, pamphlets, or other written material from your doctor or pharmacy?

Never

Rarely

Sometimes

Often

Always

Thank You!

|  | **Stool Test** | **Colonoscopy** | **CT Colonography** |
| --- | --- | --- | --- |
| **Nature of the Test** | No Preparation  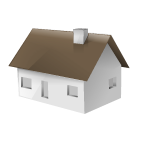  Completed at Home | *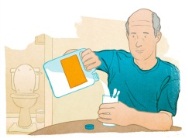  Full Day Preparation  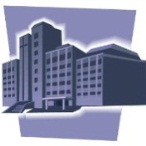  Completed at a Medical Facility | *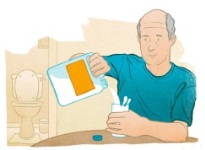  Half Day Preparation  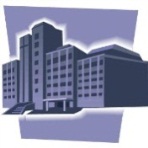  Completed at a Medical Facility |
|  |   No discomfort  No recovery Time  No Companion Required  No Travel required |   Mild-Moderate Discomfort    24 hour Recovery  *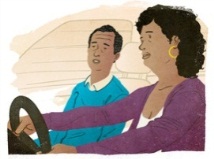  Companion Required  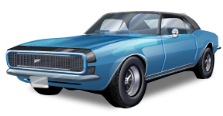  Travel to a facility |   Mild-Moderate Discomfort    1 hour Recovery  No Companion Required  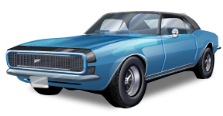  Travel to a facility |
| **Frequency** |   Every Year | 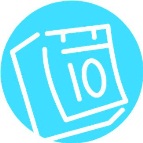  Every 10 Years | 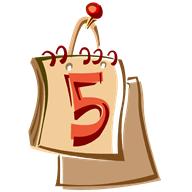  Every 5 Years |
| **Risk over 25 years of major complications** | 7 in 1000 | 8 in 1000 | 7 in 1000 |
| **Chance of needing 1 or more colonoscopies by age 65** | 62% | 100% | 46% |

*Marked Images from the National Institutes of Health Gutcheck Project http://gutcheck.nci.nih.gov/
